# Supplementary material for: Solvation of Model Biomolecules in Choline-Aminoate Ionic Liquids: A Computational Simulation Using Polarizable Force Fields
Source: Molecules. 2024 Mar 28;29(7):1524. doi: 10.3390/molecules29071524 (PMC11013605; doi:10.3390/molecules29071524)
Supplement: Supplementary file 1 [file molecules-29-01524-s001.zip › molecules-2937382-supplementary.pdf]

molecules

MDPI

Solvation of Model Biomolecules in Choline-Aminoate ionic liquids. A computational simulation using polarizable force fields.

Stefano Russo and Enrico Bodo

Supporting Information

S1 R-factor for hairpin oligopetide

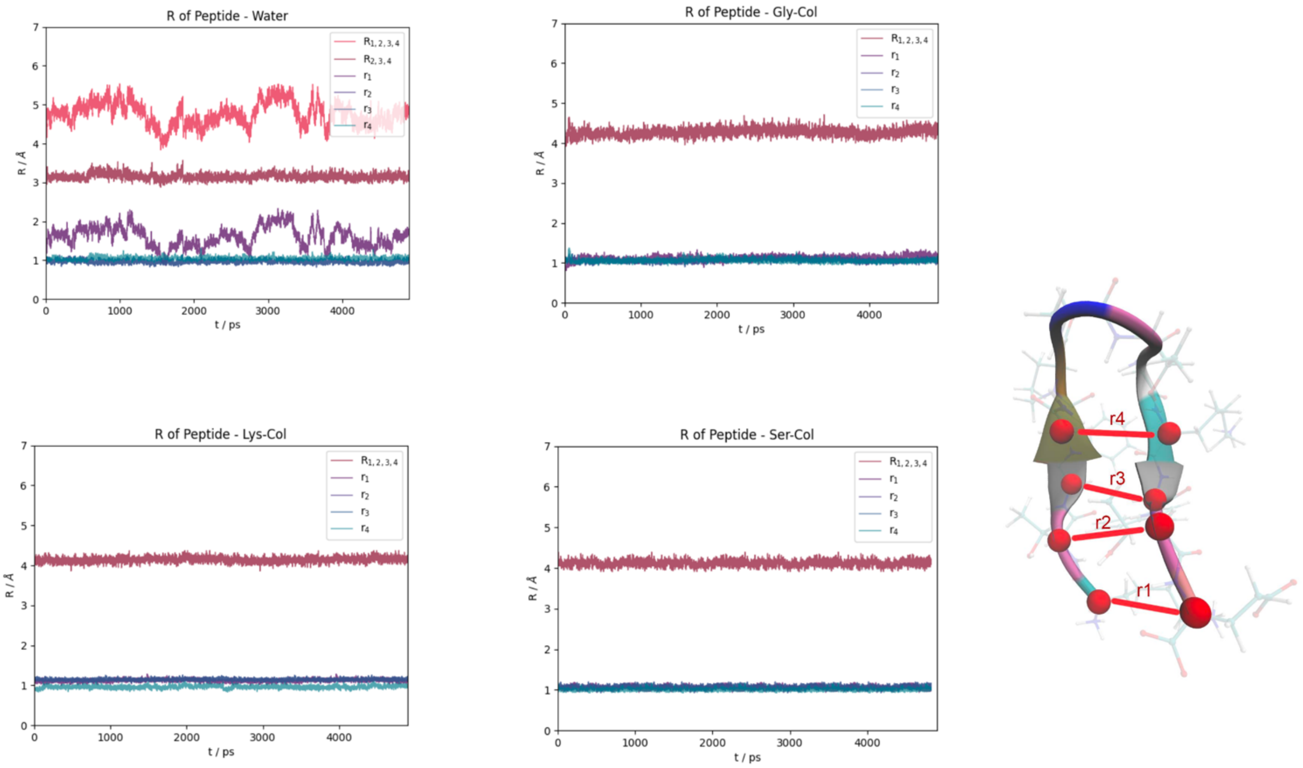

Figure S1: R factors computed along the dynamics in the first 5 ns for the 4 solvents. The r_i labels identify the ratios of the distance of two residues facing each other on the different strands with its value in the reference structure. The R factor is the sum of these ratios. A scheme of the protein including the 4 distance is reported on the right.

S2 Details of oligopetide-IL interactions

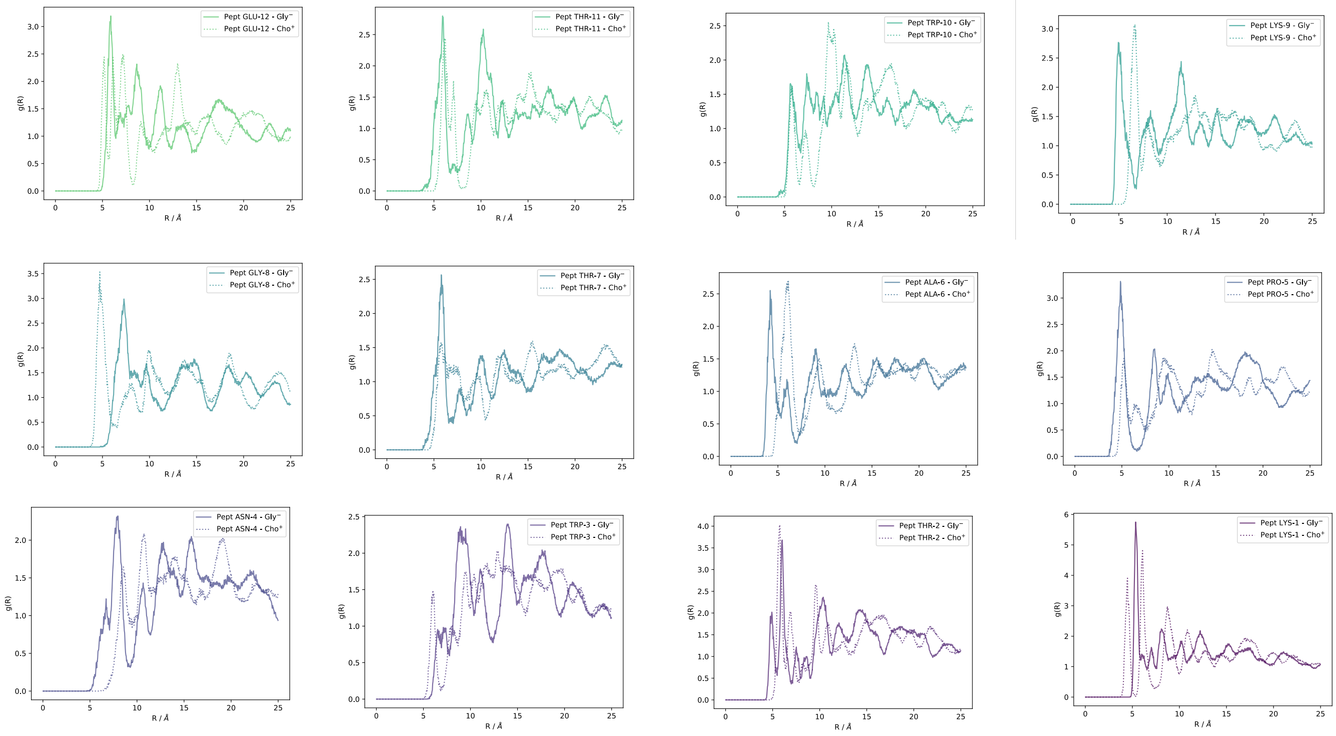

Figure S2: [Ch][Gly]: radial distribution functions between the c.o.m. of the molecular ions and the 12 residues in the hairpin.

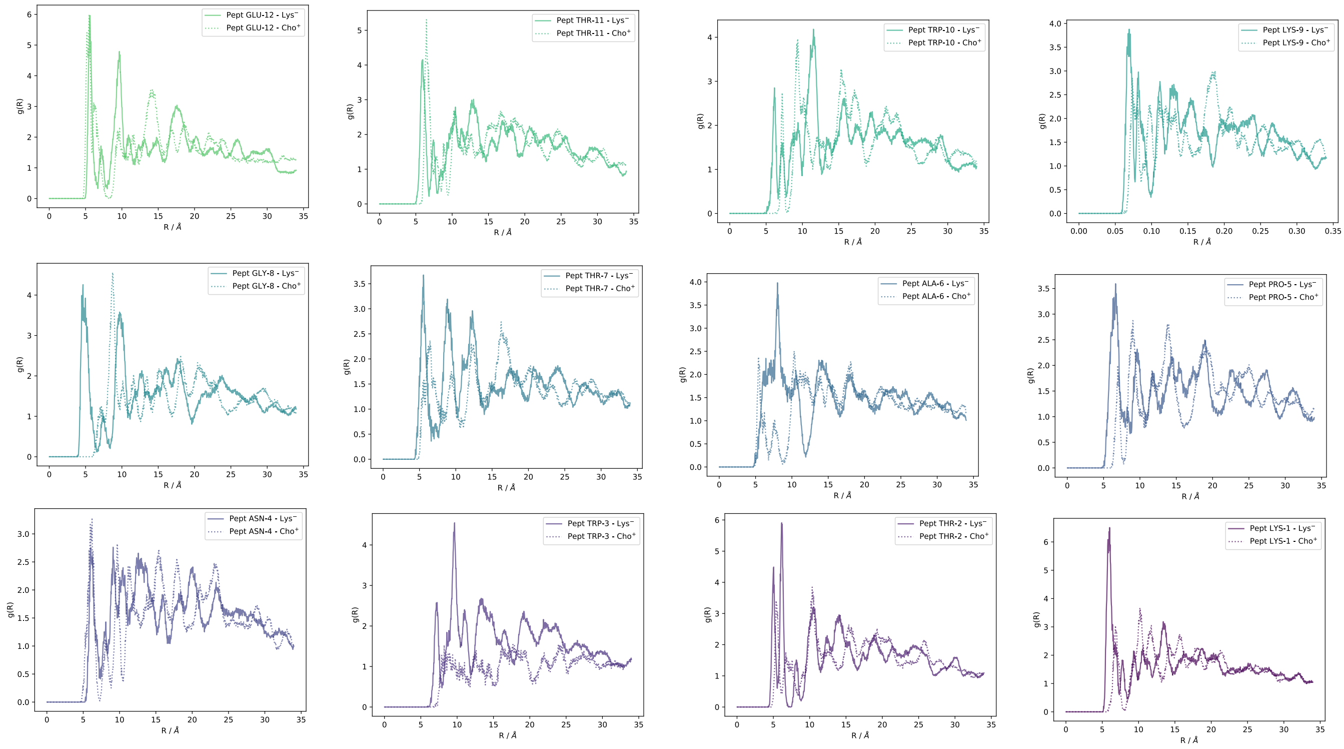

Figure S3: [Ch][Lys]: radial distribution functions between the c.o.m. of the molecular ions and the 12 residues in the hairpin.

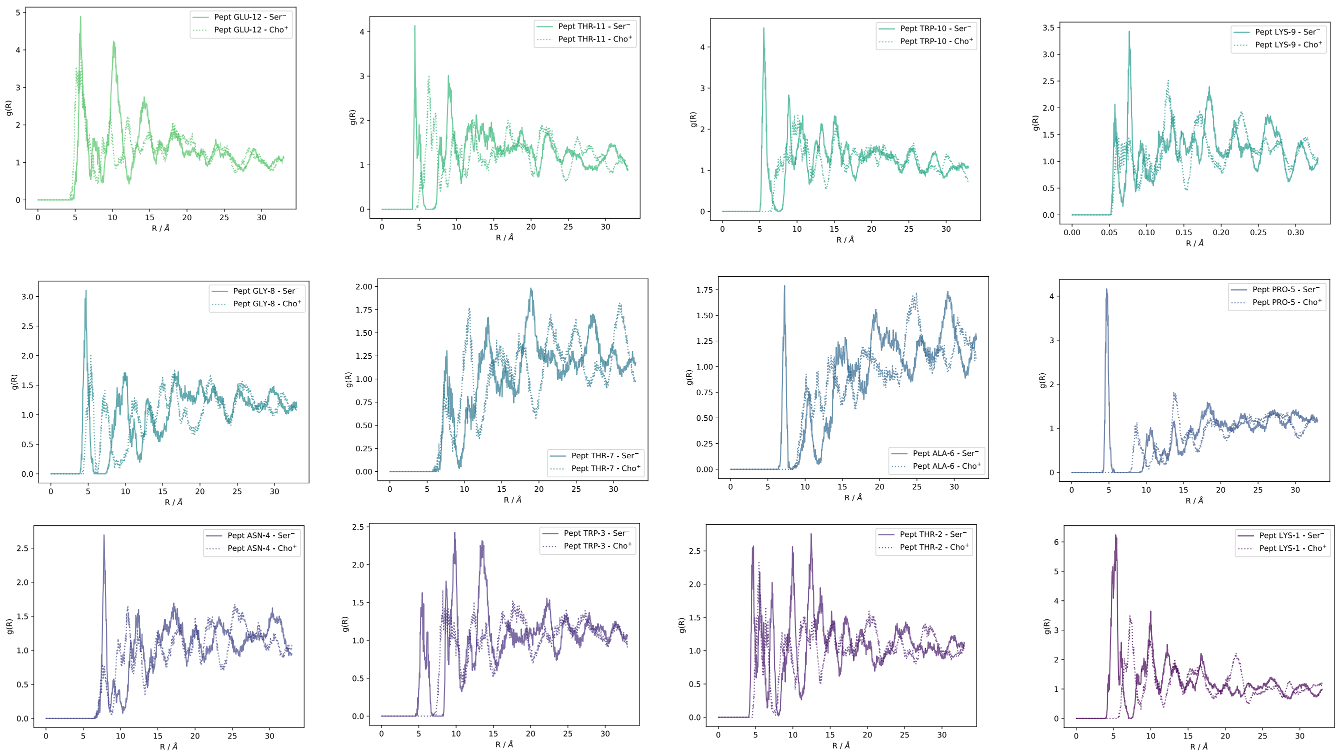

Figure S4: [Ch][Ser]: radial distribution functions between the c.o.m. of the molecular ions and the 12 residues in the hairpin.

S3 Solvation of DNA oligonucleotide

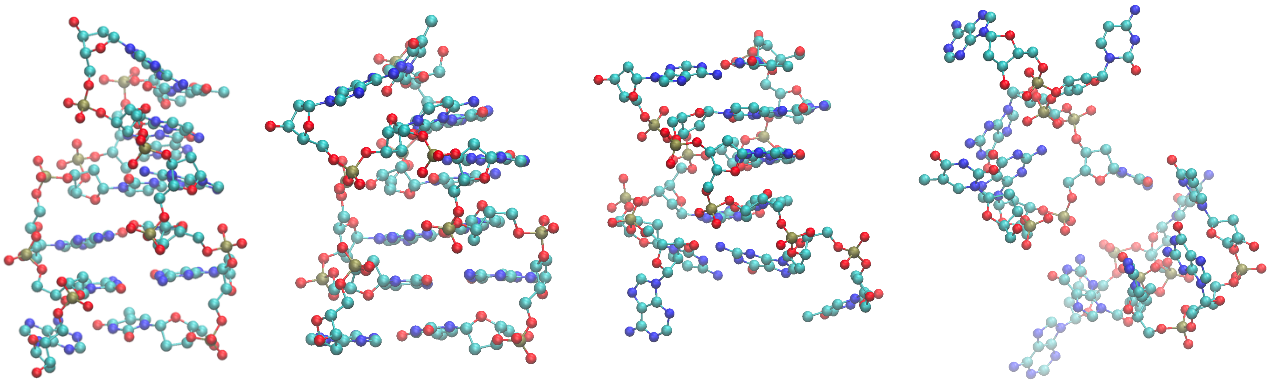

Figure S5: final snapshot of the simulations of the DNA model. From left to right the solvents are: water, [Ch][Lys], [Ch][Ser], [Ch][Gly]. The solvent is not shown for clarity.

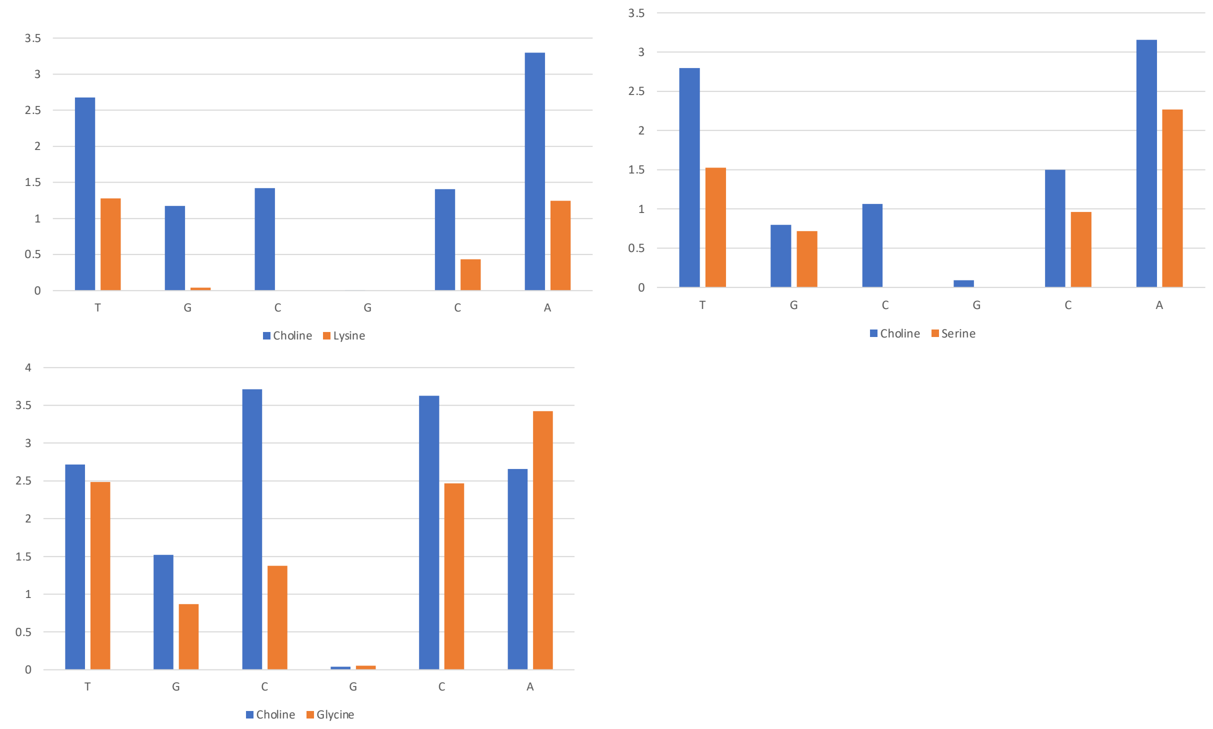

Figure S6: Composition of the solvent layer surroundings of the DNA model. In reading order: [Ch][Lys], [Ch][Ser] and [Ch][Gly]. The histogram reports the average number of cations (blue) and anions (orange) within 7 Angstroms of the c.o.m. of the base (excluding phosphate and ribose).

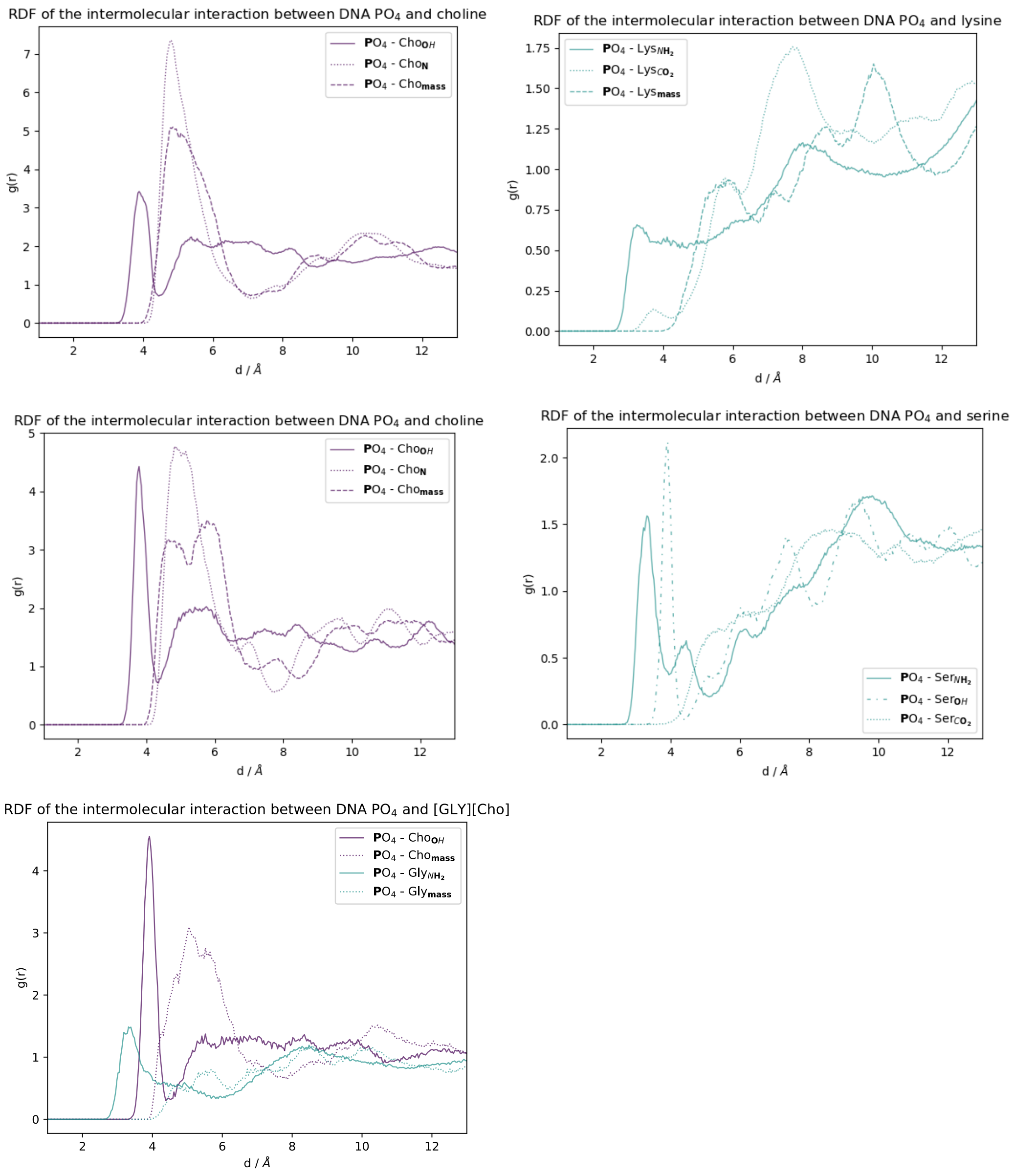

Figure S7: RDFs calculated between the c.o.m. of anions and cations of the [Ch][AA] liquid and the c.o.m. of the DNA phosphate.

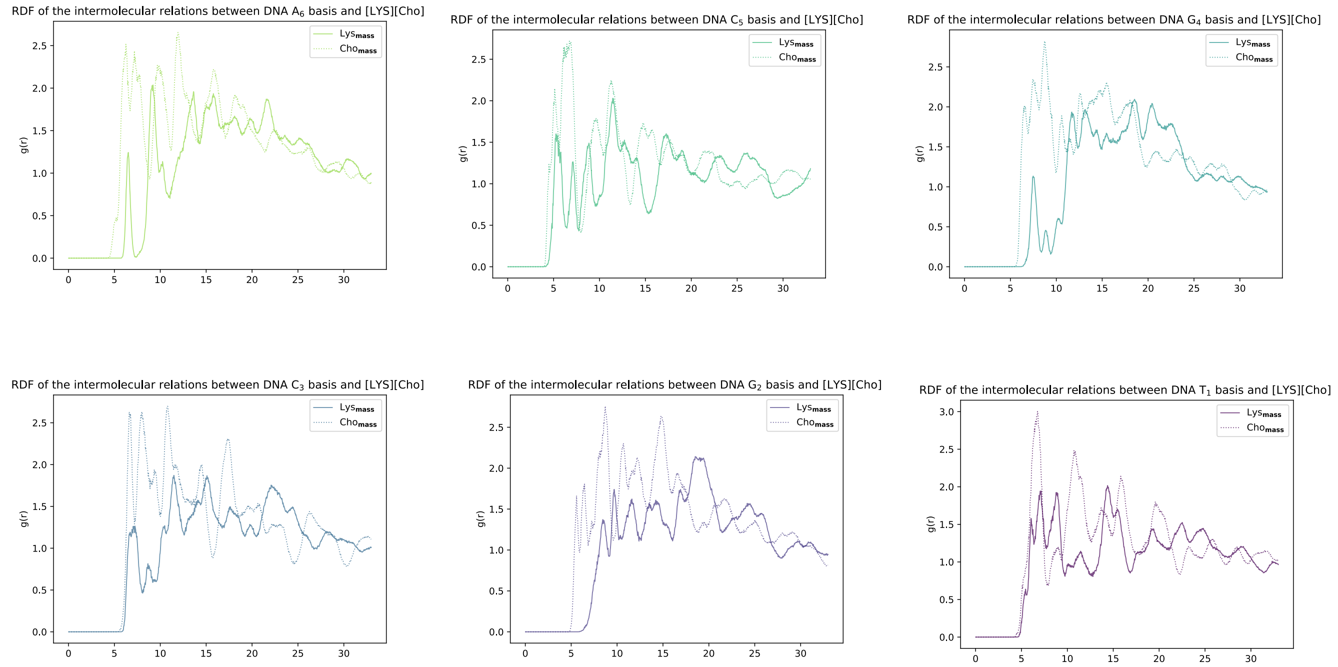

Figure S8: RDFs calculated between the c.o.m of the anion/cation of the [Ch][Lys] and the specific DNA base.

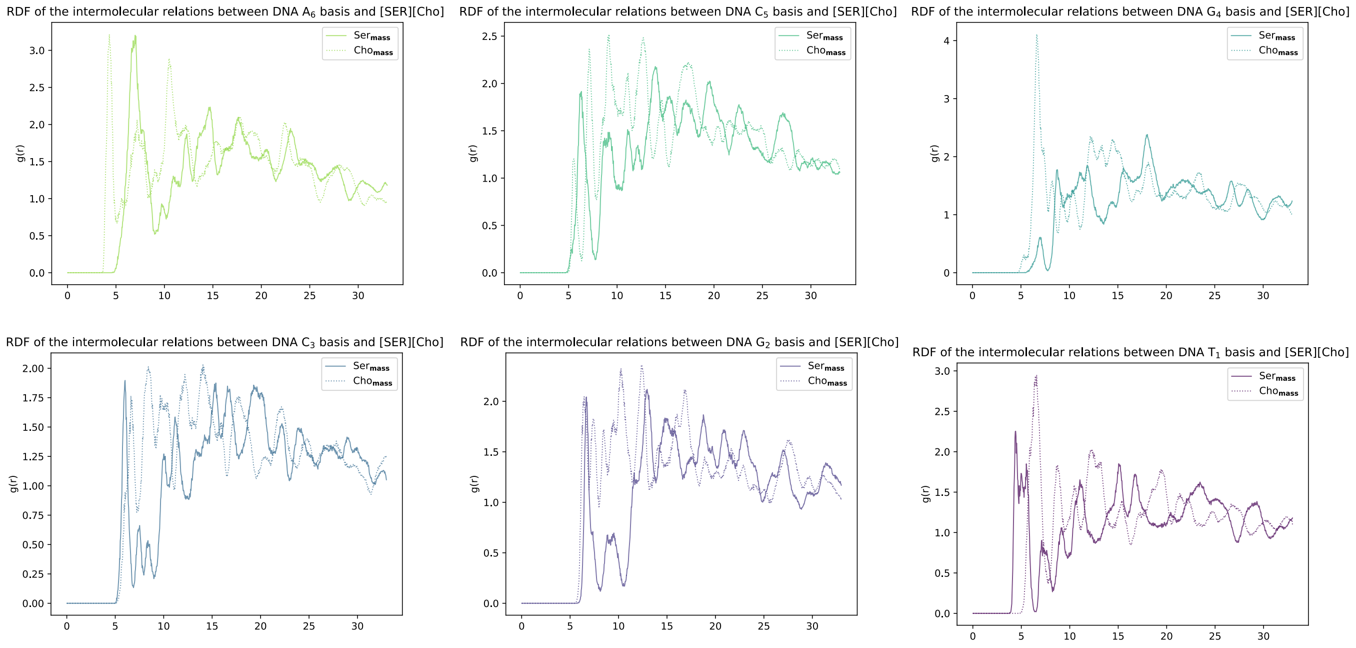

Figure S9: RDFs calculated between the c.o.m of the anion/cation of the [Ch][Ser] and the specific DNA base.

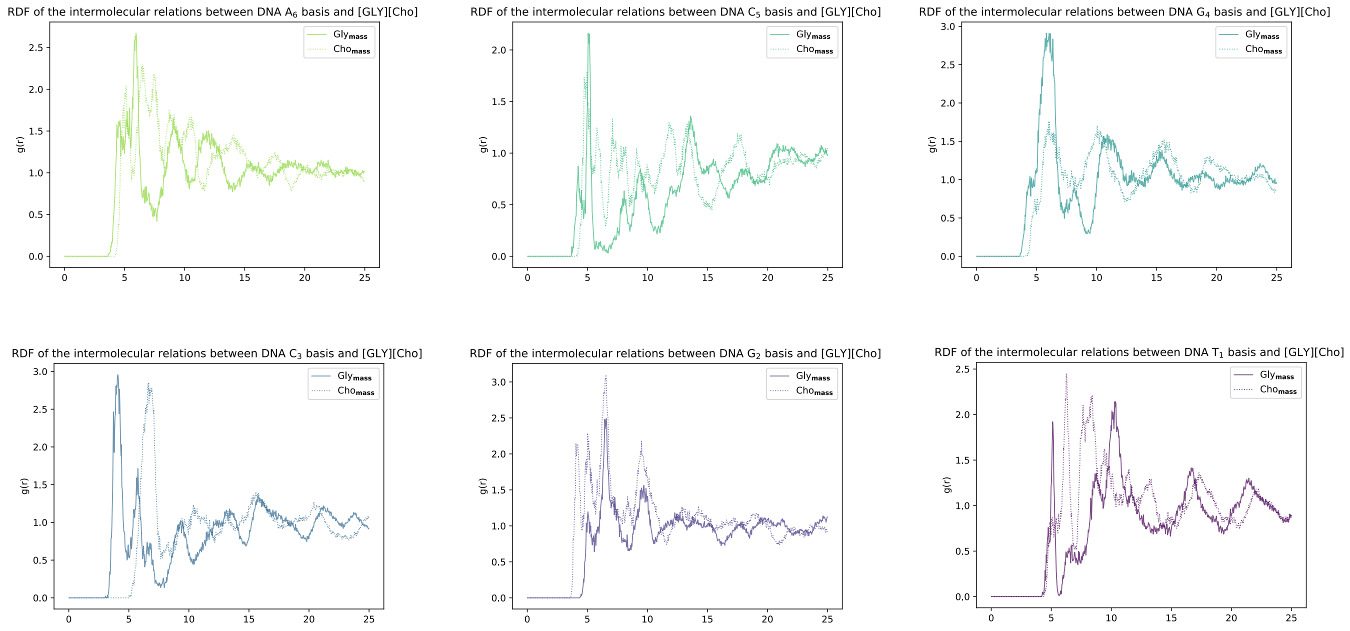

Figure S10: RDFs calculated between the c.o.m of the anion/cation of the [Ch][Gly] and the specific DNA base.

S4 Validation of the force field

Validation of the force field for the neat [Ch][AA] liquids has already been presented in ref. [51] in great details. Here we limit ourselves to showing how the ILs force field is compatible with the parametrization in the *AMOEBA-pro/nuc* one. The simplest way to provide an assessment of the quality of a polarizable force field, is to compare its performance against ab-initio data. Specifically, we have taken a suitably saturated Asn, Trp, adenosine (A) and thymidine (T) (all in their neutral form) and compared the interaction energies provided by our force field with DFT ab-initio data. The computational approach closely follows the one proposed in ref. [65] where the starting minimum geometry of 16 complexes (namely X-cholinium, X-Lysinate, X-Serinate and X-glycinate, with X=Asn, Trp, A, T) has been obtained using *AMOEBA* and a subsequent scan along the distances between the two molecules has then been performed keeping the two molecular partners rigid. Once identified a set of suitable geometries along the scan, an ab-initio evaluation and decomposition of the total interaction energy along the them has been performed using the Psi4 code [66] and its sSSAPT0 [67] module with the 6-311G* basis set. In SAPT the total interaction energy is decomposed into various terms, among which the electrostatic and dispersion contributions can be easily compared to their force field analogues. Results are displayed in Figures S11-18. The agreement between the two sets of data is remarkable, especially considering that the parameters describing the ILs have been developed independently from those in the *AMOEBA* used for the biomolecule fragment. The match between ab-initio and *AMOEBA* is excellent for all the X-aminoate complexes where differences are at most 10% (the force field predicts more negative interaction energy) because of the force field underestimating the repulsive short-range potential. The force field electrostatic is, however, remarkably similar to the ab-initio one for all aminoate complexes.

A slightly larger discrepancy is found in the data of the X-cholinium interaction (with a maximum for Ch-Trp, Figure S13) where the force field underestimate electrostatic interaction (i.e. the force field electrostatic is less negative). This effect is however mitigated by the mentioned tendency of the force field to underestimate repulsion (where the force field provide less positive energy). For Ch-Asn and Ch-Trp, the disagreement between force field and the ab-initio energy is larger than that we have found for Ch-A and Ch-T.

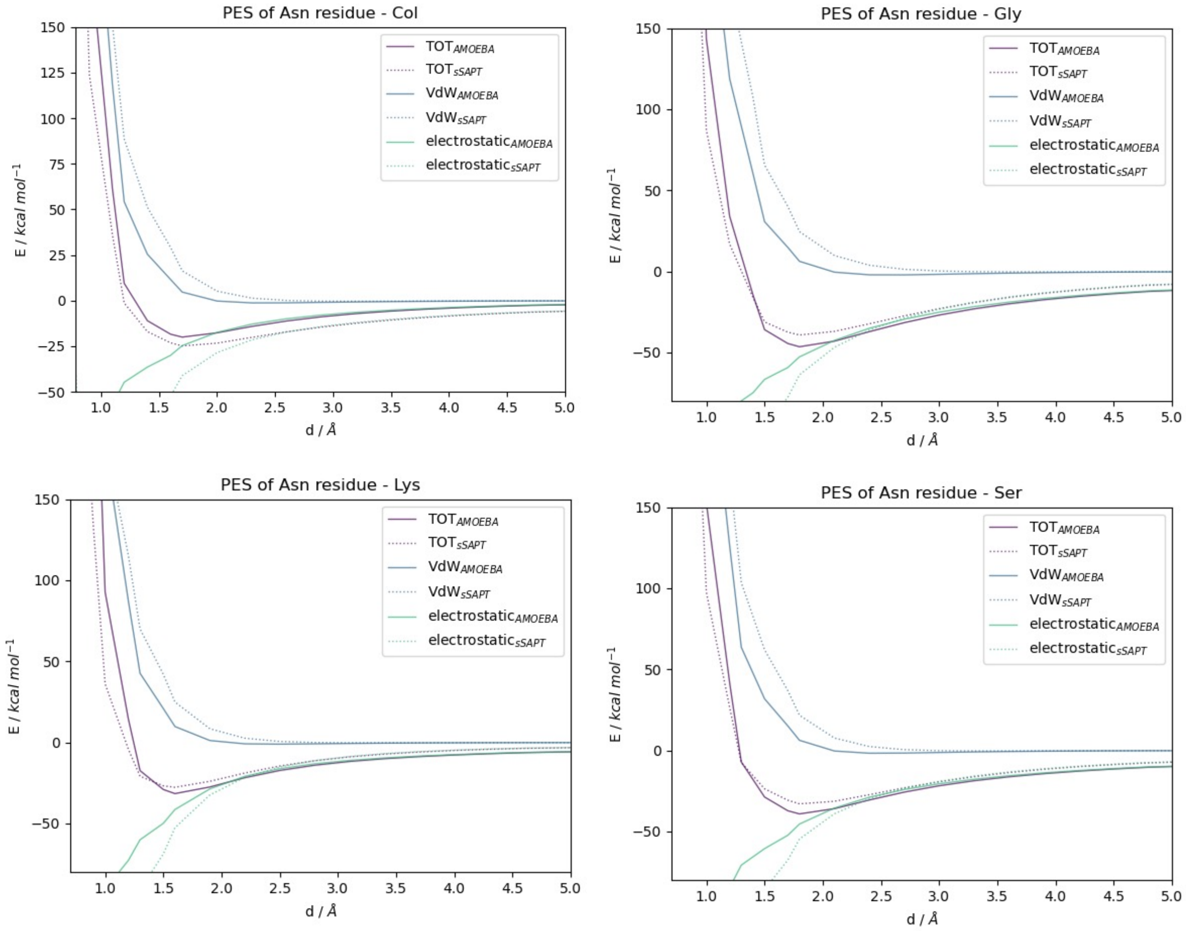

Figure S11: Energies along rigid scan between an Asn residue (neutral) and the corresponding molecular ion. The violet curves are for total energies, the blue ones for the dispersion energy and the green ones for electrostatic.

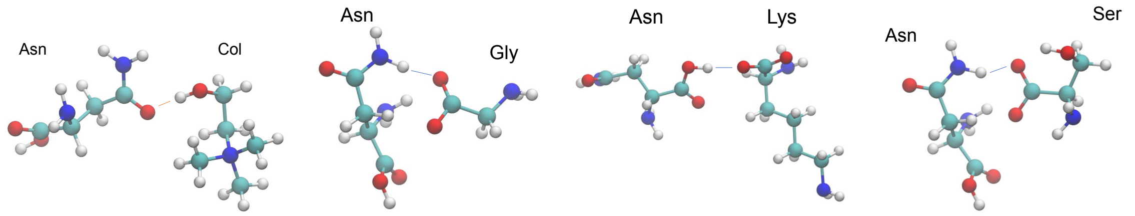

Figure S12: minimum geometries along the scans of Figure S11. The distances used as x-axis in Figure S11 is also indicated.

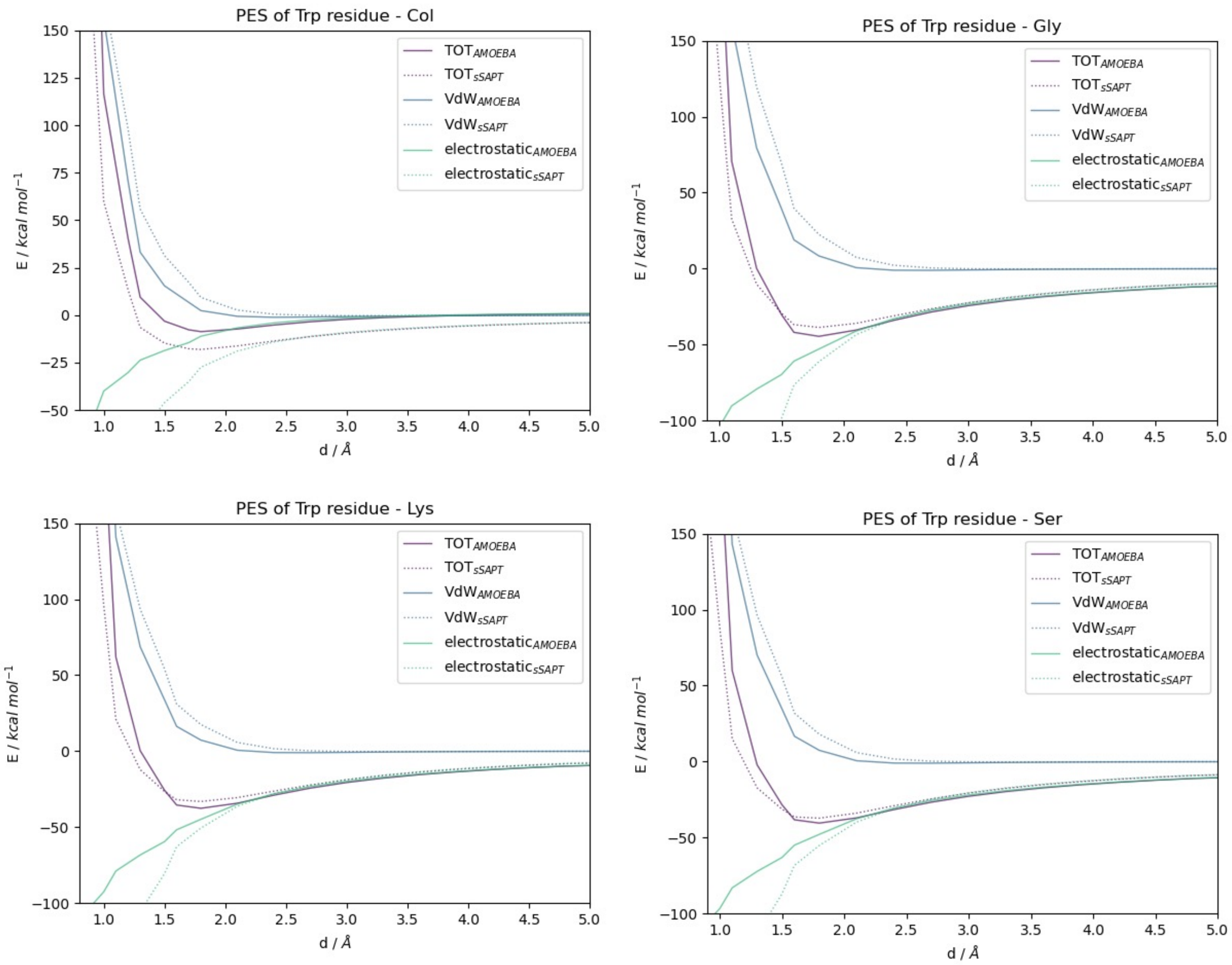

Figure S13: Energies along rigid scan between a Trp residue (neutral) and the corresponding molecular ion. The violet curves are for total energies, the blue ones for the dispersion energy and the green ones for electrostatic.

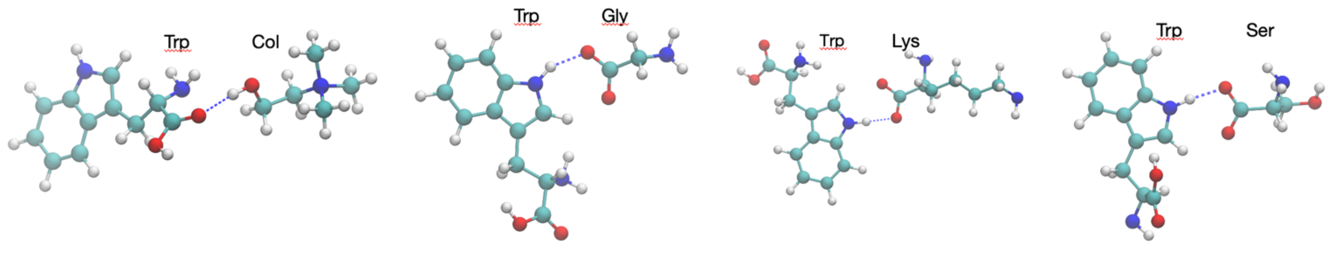

Figure S14: Minimum geometries along the scans of Figure S13. The distances used as x-axis in Figure S13 is also indicated

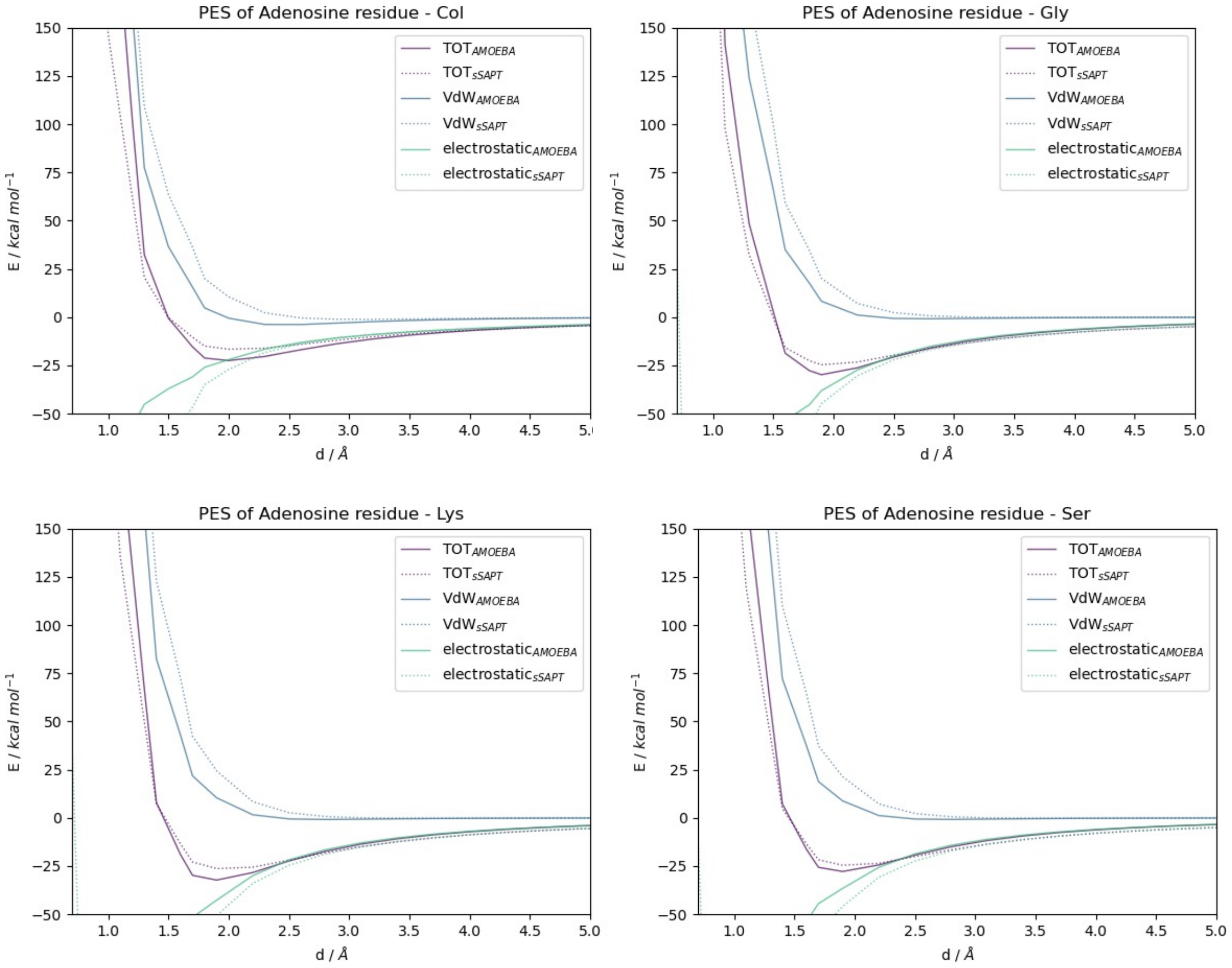

Figure S15. Energies along rigid scan between an adenosine and the corresponding molecular ion. The violet curves are for total energies, the blue ones for the dispersion energy and the green ones for electrostatic.

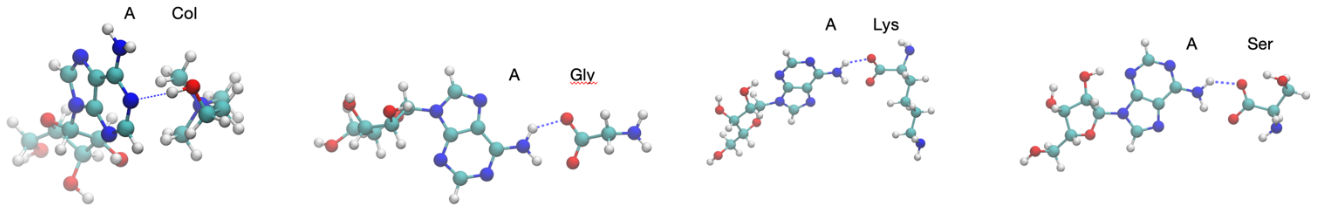

Figure S16. minimum geometries along the scans of Figure S15. The distances used as x-axis in Figure S15 is also indicated.

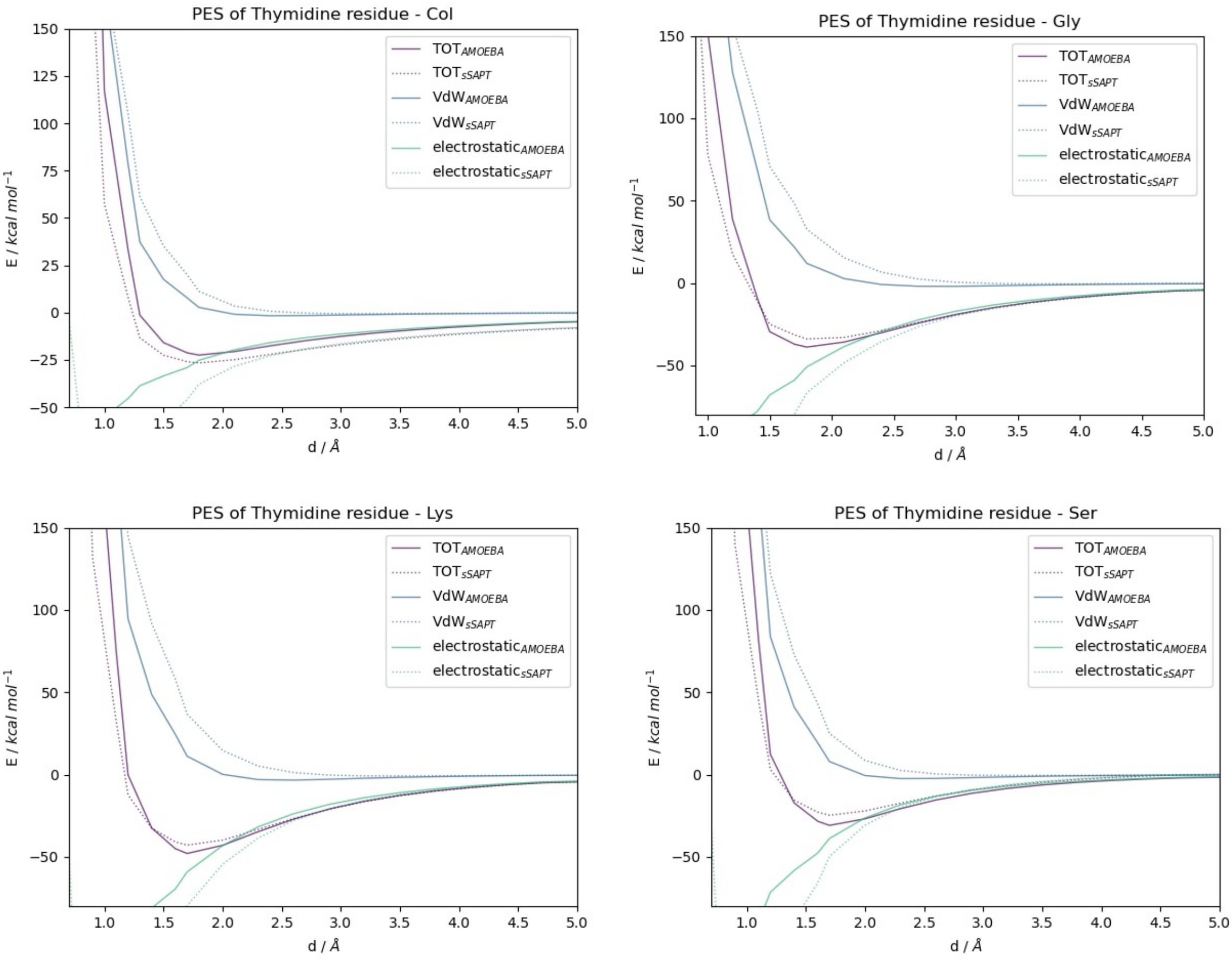

Figure S17. Energies along rigid scan between a thymidine and the corresponding molecular ion. The violet curves are for total energies, the blue ones for the dispersion energy and the green ones for electrostatic.

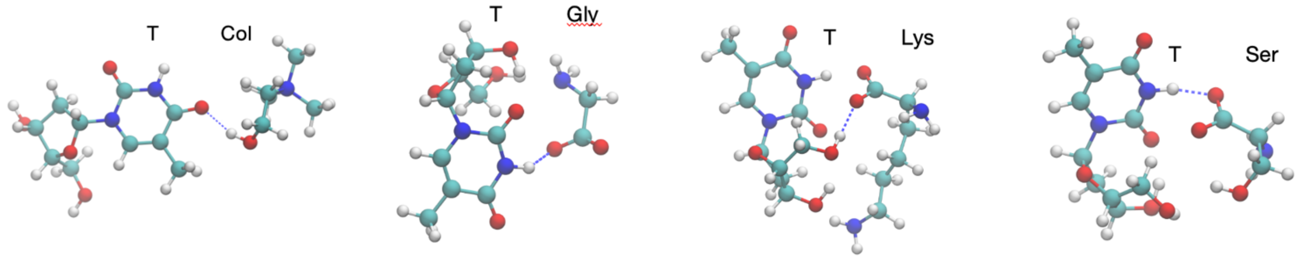

Figure S18. Minimum geometries along the scans of Figure S17. The distances used as x-axis in Figure S17 is also indicated.

S5 Simulations details

- Thermostat Bussi-Parrinello: τ=0.1ps
- Barostat Berendsen: τ=2ps
- VdW-cutoff: 10 Å
- Ewald-cutoff: 10 Å
- Particle Mesh Ewald grid: 200 200 200

	Time-step (fs)	Save-time (ps)	Production time (ns)
		**Peptide**	
**Water**	1.0	0.1	12
**Glycinate**	0.5	0.1	7
**Lysinate**	0.5	0.1	5
**Serinate**	0.5	0.1	5.7
		**DNA**	
**Water**	0.5	0.1	7.6
**Glycinate**	0.5	0.1	6
**Lysinate**	1.0	0.1	14
**Serinate**	1.0	0.1	10
